# A lncRNA survey finds increases in neuroprotective LINC‐PINT in Parkinson’s disease *substantia nigra*

**DOI:** 10.1111/acel.13115

**Published:** 2020-02-20

**Authors:** Alon Simchovitz, Mor Hanan, Nadav Yayon, Songhua Lee, Estelle R. Bennett, David S. Greenberg, Sebastian Kadener, Hermona Soreq

**Affiliations:** ^1^ The Department of Biological Chemistry and The Edmond and Lily Safra Center for Brain Sciences The Hebrew University of Jerusalem Jerusalem Israel; ^2^ Biology Department Brandeis University Waltham MA USA

**Keywords:** Alzheimer's disease, LINC‐PINT, lncRNA, neurodegeneration, Parkinson's disease, RNA‐Seq

## Abstract

Recent reports highlight regulatory functions of long noncoding RNAs (lncRNAs) in neurodegeneration and aging, but biomedical implications remain limited. Here, we report an rRNA‐depletion‐based long RNA‐Sequencing Resource of 65 substantia nigra, amygdala, and medial temporal gyrus samples from Parkinson's disease (PD) and matched control brains. Using a lncRNA‐focused analysis approach to identify functionally important transcripts, we discovered and prioritized many lncRNAs dysregulated in PD. Those included pronounced elevation of the P53‐induced noncoding transcript LINC‐PINT in the substantia nigra of PD patients, as well as in additional models of oxidative stress and PD. Intriguingly, we found that LINC‐PINT is a primarily neuronal transcript which showed conspicuous increases in maturing primary culture neurons. LINC‐PINT also accumulated in several brain regions of Alzheimer's and Huntington's disease patients and decreased with healthy brain aging, suggesting a general role in aging and neurodegeneration for this lncRNA. RNAi‐mediated depletion of LINC‐PINT exacerbated the death of cultured N2A and SH‐SY5Y cells exposed to oxidative stress, highlighting a previously undiscovered neuroprotective role for this tumor‐inducible lncRNA in the brains of patients with neurodegenerative disorders.

## Graphical abstract

We performed RNA‐Seq on a large cohort of brain tissues from Parkinson's disease patients and controls. Next, we applied a novel lncRNA‐based analysis method to the data to screen for functionally relevant lncRNAs, identifying LINC‐PINT. Comparison to other models identified LINC‐PINT as a neuronal transcript dysregulated in additional neurodegenerative diseases and in brain aging. Finally, functional analysis revealed LINC‐PINT to be neuroprotective in the context of oxidative stress.

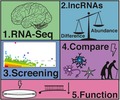

## 1 INTRODUCTION

Parkinson's disease (PD) is the second most common neurodegenerative disorder, affecting approximately 2% of adults over the age of 70 worldwide (Pringsheim, Jette, Frolkis, & Steeves, 2014). PD involves the progressive and selective death of dopaminergic neurons in the *substantia nigra pars compacta*, hampering the nigrostriatal pathway and inducing bradykinesia, gait abnormalities, resting tremor, and cognitive symptoms (Simchovitz, Soreq, & Soreq, 2016). While PD is largely considered a sporadic disease, autosomal dominant or recessive mutations in several genes including alpha‐synuclein (SNCA) have been identified as inducing familial PD (Hauser & Hastings, 2013). Another risk factor associated with increased PD prevalence is environmental exposure to toxins such as the oxidative stress‐inducing herbicide Paraquat (PQ) (Bastias‐Candia, Zolezzi, & Inestrosa, 2019). However, the contributions to PD of changes in the brain's transcriptome are incompletely understood.

One class of RNAs with unclear role in PD initiation and progression includes long noncoding RNAs (lncRNAs), which are a diverse subset of transcripts longer than 200 nucleotides that do not encode for proteins. LncRNAs participate in key cellular functions such as modification of transcriptional and translational processes by various molecular mechanisms, including scaffolding of RNA–protein structures, competition with endogenous mRNAs over microRNA (miRNA) binding (Kopp & Mendell, 2018), and epigenetic regulation (Marin‐Bejar et al., 2013). LncRNAs are involved both in normal physiological functions, such as embryonic development and muscle activity, and in pathological states such as cancer and cardiovascular disease (Huarte, 2015; Zhu et al., 2018). In the healthy central nervous system, lncRNAs play a role in neuronal function and differentiation (Fatica & Bozzoni, 2014; Tan et al., 2017). Neurodegenerative states including Alzheimer's disease (AD) involve dysregulation of various lncRNAs, such as BACE1‐AS (Wan, Su, & Zhuo, 2017), indicating functional relevance in this realm as well.

The low evolutionary conservation of lncRNAs (Hezroni et al., 2015) and the contributions of primate‐specific noncoding RNAs to human‐specific functions (Barbash, Simchovitz, et al., 2017) highlight the importance of investigating lncRNAs in both human patient tissues and cell and animal models of neurodegenerative disease. However, limited tissue availability directed current transcriptome‐wide work on PD‐related lncRNAs in human patients to peripheral blood (Simchovitz et al., 2016; Soreq et al., 2014), with profiling of PD‐related lncRNAs in the human brain largely limited to predetermined subsets of lncRNAs via microarrays or qPCR (Elkouris et al., 2019; Ni et al., 2017), lacking actual transcriptome‐wide analysis. While recent research has shown that a handful of selected lncRNAs expressed in the PD brain are also expressed in peripheral blood, brain and peripheral tissues showed distinct lncRNA change patterns (Elkouris et al., 2019). The distinct levels and disease‐related changes in lncRNAs in peripheral blood and the brain may reflect their high tissue specificity (Mele et al., 2015).

In our current study, we sought global lncRNA changes in PD patients’ brains by performing deep RNA sequencing of brain tissues from the *substantia nigra*, amygdala, and medial temporal gyrus of PD patients and controls. Our analysis approach was aimed at detecting functionally important lncRNA candidates with high relevance to disease pathology and identified LINC‐PINT as a differentially expressed (DE) lncRNA in the PD brain. Focusing on the roles of LINC‐PINT, we challenged its roles in experimental cell culture tests of human and mouse neuronal origin.

## 2 RESULTS

### 2.1 RNA‐Seq demonstrates substantial transcriptional changes in the PD *substantia nigra*

**Figure 1:**
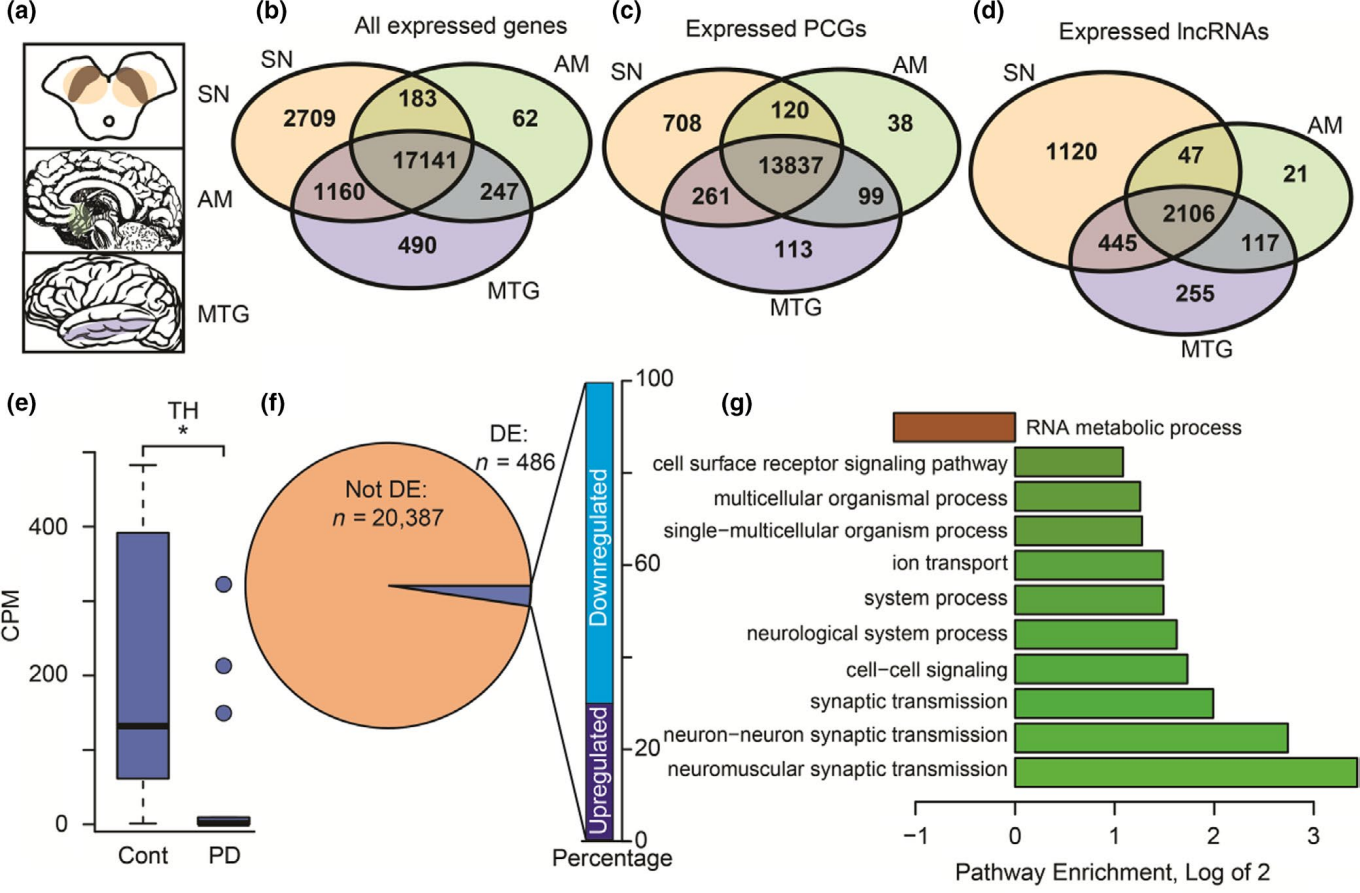
Brain tissues from Parkinson's disease patients display distinct disease‐related changes in gene expression. (a) Schematic view of the three analyzed brain regions (AM, amygdala; SN, *substantia nigra*; MTG, middle temporal gyrus). (b‐d) Venn diagram of expressed genes in the analyzed brain regions, with separation to (c) protein‐coding genes (PCGs) and (d) lncRNAs. (e) Boxplot of tyrosine hydroxylase expression in the SN shows a decrease in PD compared to control donors (*p* < .05, statistical analysis using edgeR; outliers shown as circles). (f) Pie chart describing the number of DE genes and bar graph describing the direction of change in the SN. (g) Bar plot describing the enrichment of top‐enriched pathways as analyzed by the *Panther* algorithm (enrichment larger than 2 or smaller than 0.5, FDR‐corrected *p*‐value for enrichment < .001)

To pursue PD‐related lncRNAs, we generated ribosomal‐RNA‐depleted RNA‐Seq libraries from 75 *substantia nigra* (SN), amygdala (AM), and medial temporal gyrus (MTG) tissues (Figure 1a) of PD patients and nondemented control donors from the Netherlands Brain Bank using the RNATagSeq protocol (Shishkin et al., 2015) (see Table S1 for full information on the patients and samples). To limit the effects of high inter‐individual variability in RNA quality on library accuracy, we sequenced only those samples with RNA integrity numbers (RIN) above 6.5, yet used both those and the rest of the samples for consequent qPCR validations. To avoid RNATagSeq‐mediated bias in library size due to variable adapter affinity, we excluded 10 libraries with fewer than 500,000 annotated reads from analysis. The remaining 65 libraries (23 SN, 19 AM, 23 MTG, parameters detailed in Table 1 and Figure S1; raw data accessible at the GEO as GSE114517) were analyzed separately per tissue using the edgeR algorithm (Robinson, McCarthy, & Smyth, 2010), accounting for age and sex to correct for biological variance, and for RIN values to correct for technical variance.

**Table 1:** Demographic and technical parameters of RNA‐Seq samples included in analysis

	Sample number		Percent of male subjects		Age (mean ± *SD*)		RIN (mean ± *SD*)	
	PD	Cont	PD	Cont	PD	Cont	PD	Cont
AM	15	8	86	50	74.9 (±1.8)	86.8 (±2.0)	7.1 (±0.2)	7.3 (±0.3)
MTG	13	6	77	50	76.2 (±2.1)	85.8 (±4.8)	7.1 (±0.2)	7.2 (±0.2)
SN	14	9	71	44.44	75 (±1.7)	86 (±2.0)	7.4 (±0.2)	7.38 (±0.2)

Note

Data are presented as numbers for sample number (columns 1 and 2), percentage of males for sex (columns 3 and 4), and means (±*SD*) for age and RIN (columns 5–8).

The extensive histological changes in the PD SN include depletion of dopaminergic neurons and accumulation of other cell types (Fearnley & Lees, 1991). Therefore, differential expression (DE) of genes in the SN could be attributed to changes in tissue composition, especially in dopaminergic neurons. To avoid such false‐positive results, we added compensation elements for astrocytes, microglia, and dopaminergic cells (See Data S1 and Figure S2). Importantly, since such corrections may introduce bias of their own, we later tested our main results in the uncorrected model as well, as detailed below.

We identified 21,193, 17,633, and 19,038 expressed genes in the SN, AM, and MTG (CPM > 1), respectively; those included all of the gene types in the GENCODE database (Harrow et al., 2012), with the majority of genes—17,141—predictably common to these three brain regions (Figure 1b). Separate examination of protein‐coding genes (PCGs) and lncRNAs revealed that most genes in both groups were expressed across all three brain regions (Figure 1c,d). Predictably, we observed a fourfold decline of the dopaminergic neuron marker tyrosine hydroxylase (TH) in the PD compared to the control SN prior to correction for cell‐type markers (Figure 1e). Also, 486 genes were DE between the PD and control SN after this correction (FDR < 0.05), of whom 340 were downregulated and 146 upregulated (Figure 1f; full list of DE genes in Table S2). GO term analysis (Mi et al., 2017) revealed several neuronal, and more specifically, synaptic pathways which were significantly enriched for DE genes in the PD SN (Figure 1g).

### 2.2 LncRNA‐centered search identifies disease‐induced lncRNA changes in the Parkinsonian SN

**Figure 2:**
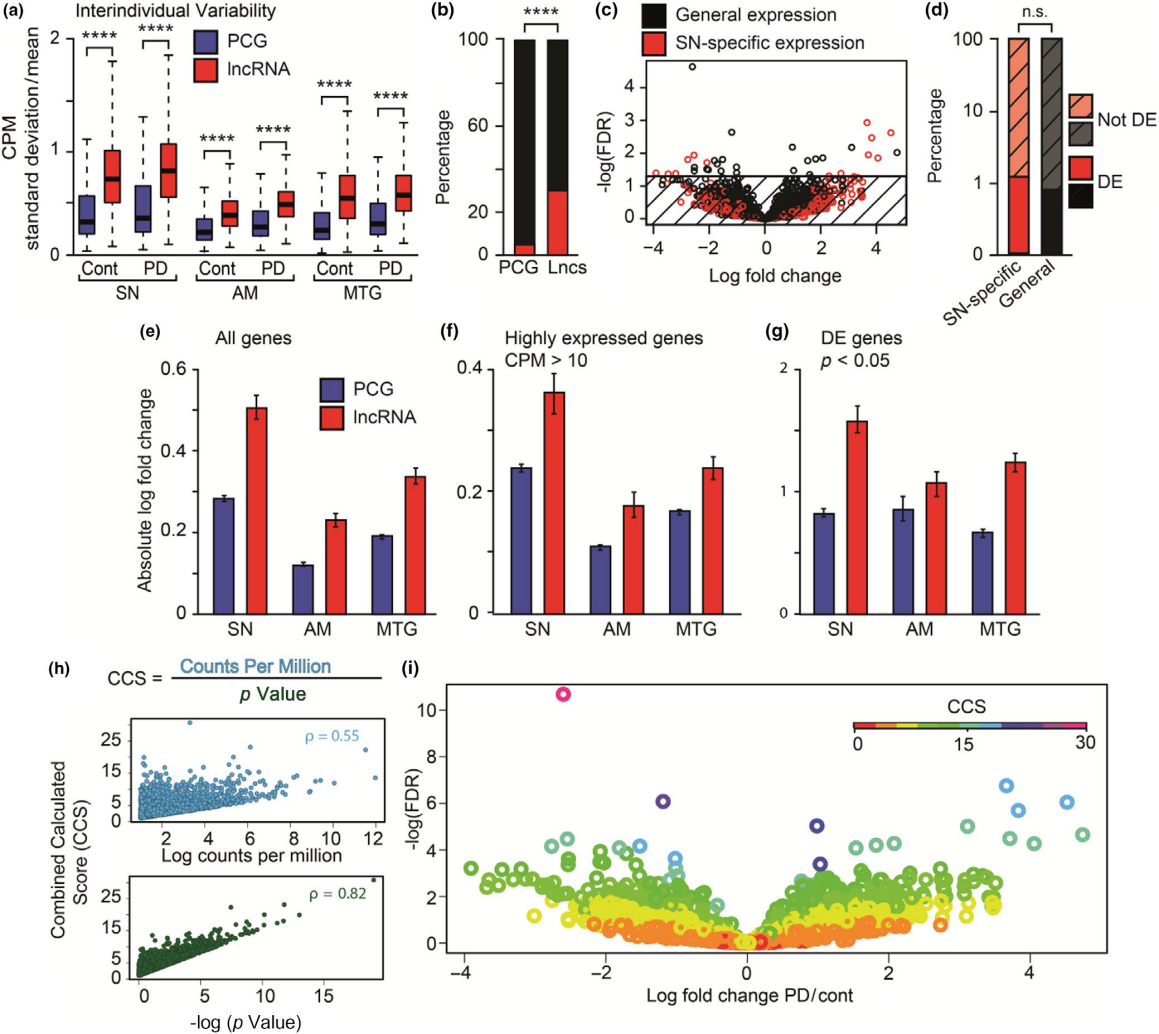
LncRNAs display distinct disease‐induced changes in PD emphasized by a novel lncRNA‐based analysis method. (a) Boxplots demonstrating larger median standard deviation for the expression of lncRNAs compared to PCGs in the SN (14 patients vs. 9 controls), AM (15 patients vs. 8 controls), and the MTG (13 patients vs. 6 controls) (*p* < .0001, Mann–Whitney U test). (b) Bar plot depicting enrichment in SN‐specific expression (red) versus general expression (black) of lncRNAs compared to PCGs (Fisher's exact test, *p* < .0001). (c) Volcano plot of SN‐specific (red) versus generally expressed (black) lncRNAs. The horizontal line marks an FDR of 0.05, indicating 33 statistically significant changes. (d) Bar plot depicting similar proportions of differential expression among SN‐specific and generally expressed lncRNAs (Fisher's exact test, *p* = .25). (e‐g) Bar plots describing the comparison of absolute log‐fold change values between lncRNAs (red) and PCGs (blue), including (e) all genes, (f) highly expressed (CPM > 10) genes, and (g) DE (edgeR *p* < .05) genes (99% CI shown, based on random resampling). (h) The SN combined calculated score (CCS) correlates with log‐transformed absolute fold change and with *p*‐value (*p* < .0001, Rho = 0.55 and 0.82, respectively, Spearman's correlation). (i) Volcano plot of SN‐expressed lncRNAs colored according to CCS

We next sought the expression patterns of lncRNAs in the PD SN compared to other PD brain regions and to the SN of nondemented donors. LncRNAs displayed higher inter‐individual variability compared to PCGs (Figure 2a), as shown by others (Kornienko et al., 2016). While most lncRNAs were expressed across the three investigated brain regions, 1,120 out of 3,718 SN‐expressed lncRNAs were SN‐specific, compared to only 708 out of 14,926 SN‐expressed protein‐coding genes (PCGs) which were uniquely expressed in the SN (Figure 2b). The majority of the SN‐specific lncRNAs showed low‐level expression, with nonsignificant changes between PD and control brains. However, a subset of 13 out of 1,120 SN‐specific expressed lncRNAs showed significant DE between PD patients and non‐PD, nondemented controls (Figure 2c), in a proportion similar to that of generally expressed lncRNAs (Figure 2d). Those SN‐specific lncRNAs which were DE in PD brains are therefore potentially important.

Comparing PD with control samples revealed higher DE among lncRNAs compared to PCGs (Figure 2e). This effect was heightened when examining only genes which were relatively highly expressed (CPM > 10; Figure 2f) or DE (*p*‐values < .05; Figure 2g), excluding low expression levels or highly variable expression patterns of nonchanged genes as cause for these differences. Intriguingly, none of those SN PCGs or lncRNAs were identified as DE in all three brain regions, further marking the uniqueness of the SN transcriptome. Combined, these findings suggest an important role for lncRNAs in PD in general, and specifically in the SN.

Notably, the traditional and most common method used to identify disease‐related candidates in RNA‐Seq data relies solely on the statistical significance of the difference between groups, expressed as the *p*‐value of the comparison. However, this method may create a bias against detection of biologically significant DE lncRNAs due to two main reasons. First, many lncRNAs are expressed at very low levels, deeming their biological significance questionable regardless of the statistical significance of their differential expression (Kopp & Mendell, 2018). Second, lncRNAs tend to show higher inter‐individual variability (see above), leading to higher, less statistically significant *p*‐values, which again is irrelevant for their biological significance.

To overcome these obstacles and improve our capability to identify functionally relevant lncRNAs, we extended our previous methodology for scoring the statistical and biological significance of the differential expression of lncRNAs (Guffanti, Simchovitz, & Soreq, 2014). Briefly, we combined two logarithmic values: the average expression level (evaluated by CPM) and the *p*‐value for disease‐induced differential expression, to yield a combined calculated score (CCS) that corrects for the above‐mentioned limitations (see Data S1). To test whether the CCS can indeed identify disease‐related lncRNAs, we used it to interrogate four web‐available datasets (WADs) of neuronal activity (GSE93682) (Ding et al., 2017), autism (GSE59288) (Liu et al., 2016), colorectal cancer (GSE95132, paper unpublished), and atherosclerosis (GSE87534, paper unpublished). First, we identified lncRNAs with the highest CCS that survived FDR correction but that did not rank highly by FDR alone; then, we searched for them in the literature. In several cases, this highlighted clinical relevance for lncRNAs which, using traditional FDR‐based discovery methods would have been neglected yet are evidently disease‐associated (Data summary and references in Table S3). The CCS score was hence validated as a reliable way to identify disease‐associated lncRNAs at large (see Data S1 and Figure S3 for further validation steps of this analysis approach). Taken together, this indicated that our CCS analysis can rectify some of the current problems in identifying novel and relevant lncRNAs in RNA‐Seq datasets.

In our PD SN dataset, the CCS predictably correlated both to CPM and to *p*‐value (Figure 2h). This approach identified several lncRNAs which were DE with high statistical significance as unlikely to be biologically relevant according to their CCS, yet found other lncRNAs to be potentially biologically important based on their CCS, although not on their FDR values alone (Figure 2i; see Table S4 for a full list of lncRNAs and their scores).

### 2.3 Identifying functionally important DE lncRNAs in the Parkinsonian SN

**Figure 3:**
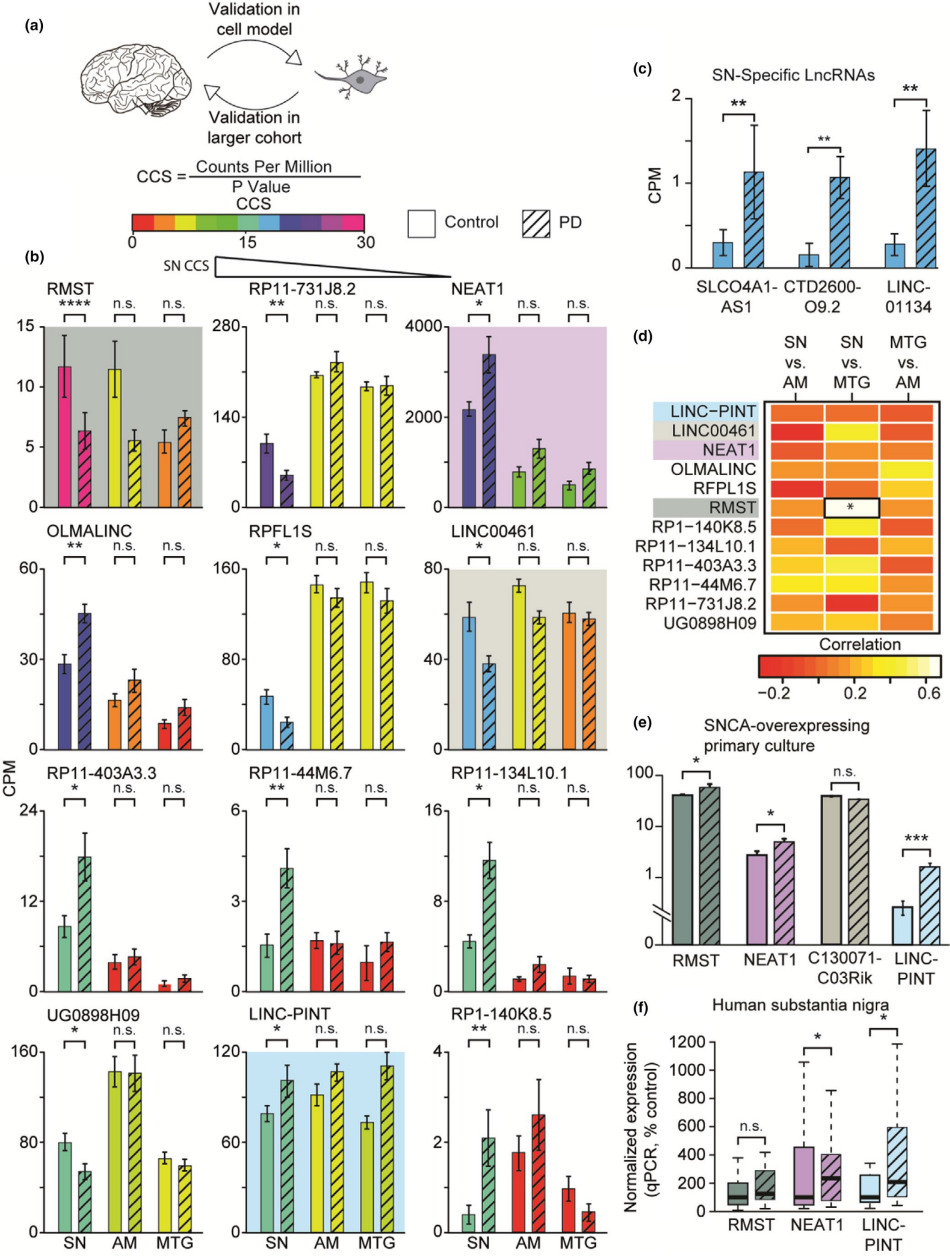
CCS‐based screen of brain‐expressed lncRNAs identifies prominent elevation of LINC‐PINT in PD SN and a primary culture model. (a) Identified lncRNAs were investigated in an additional model, and remaining candidates were subjected to investigation using qPCR on a larger cohort of SN samples. (b‐c) Bar plots of CPM values for 15 top‐scoring lncRNAs in the SN, AM, and MTG. Bars are colored according to tissue‐specific CCS. Each graph reflects a different lncRNA, shown according to decreasing score. Colored panels describe genes with murine homologs, later analyzed in panel (e); panel (c) describes lncRNAs only expressed in the SN (solid bars: controls, striped bars: PD; FDR‐corrected *p*‐values for edgeR RNA‐Seq analysis). (d) Heat map describing the correlation between the expression levels of top‐scoring lncRNAs in the three examined tissues, in a subset of 13 donors for whom all three tissues were available. Cells surrounded by a black box represent a statistically significant correlation. (e) Bar plots of SN‐top‐scoring lncRNAs which are expressed in a PD model of SNCA‐overexpressing MPNCs (GSE70368), (edgeR statistical analysis, significance not corrected for multiple comparisons; C130071C03Rik is a murine homolog of LINC00461). (f) mRNA levels for three SN and murine model DE lncRNAs in a larger cohort of human SN (control *n* = 24; PD *n* = 29), determined by qPCR (RMST— not significant; NEAT1 and LINC‐PINT—*p* < .05; Mann–Whitney single‐tail U test; solid bars: controls, striped bars: PD; % median of control group)

To limit the detection of statistically insignificant lncRNAs, we selected 15 high‐scoring lncRNAs with FDR‐corrected *p*‐value < .1 for validation in additional models and in a larger cohort (Scheme in Figure 3a). These were chosen from 90 lncRNAs overall that had FDR‐corrected *p*‐value < .1. This set included both lncRNAs that were generally expressed (in all three tissues), including rhabdomyosarcoma 2‐associated transcript (RMST) (Figure 3b), and lncRNAs expressed specifically in the SN, such as solute carrier organic anion transporter family member 4A1 antisense‐1 (SLCO4A1‐AS1) (Figure 3c). Notably, none of these SN‐modified lncRNAs showed a significant PD‐related change in the AM or MTG or scored highly in those tissues (Figure 3b). To seek correlations between the expression patterns of those top‐scoring lncRNAs in different brain regions, we examined a subset of 13 donors for whom all three examined brain regions were available (39 libraries overall), and identified only a single statistically significant correlation, between RMST expression levels in the SN and MTG (Figure 3d). This further marked the importance of investigating the transcriptional landscape of the SN*.*

Next, we considered the possibility that the observed lncRNA changes reflect accidental findings. First of all, we checked whether additional brain pathologies could affect lncRNA expression. We re‐performed the differential analysis of the SN using past occurrence of cerebrovascular accident as a confounding factor and observed no such effect neither a change in our final findings for these top lncRNAs (Figure S4). To further challenge the robustness of our findings, we selected 4 of the top 15 lncRNAs for whom murine homologs have been identified, and tested for their expression in murine neuronal primary culture (MPNC) cells overexpressing the PD‐causing gene SNCA (GSE70368) (Volakakis et al., 2015). Supporting our CCS method, three of these lncRNAs were DE in this model as well—RMST, Nuclear Enriched Assembly Transcript 1 (NEAT1), and P53‐Induced Noncoding Transcript (LINC‐PINT) (Figure 3e). To corroborate our results using a different method, we performed qPCR on a larger cohort of human SN samples (control: *n* = 24; PD: *n* = 29), using primers targeting the DE lncRNAs RMST, NEAT1, and LINC‐PINT. This larger validation cohort revealed consistent elevation in both PD and PD models of NEAT1, which has been implicated in Huntington's disease (HD) and in murine PD models (Liu & Lu, 2018) as well as LINC‐PINT, but RMST’s qPCR levels showed no disease‐related changes (Figure 3f). Therefore, we next focused on the PD‐related changes in LINC‐PINT.

LINC‐PINT presented a statistically significant (*p* < .01) 2.2‐fold increase in the PD versus control SN but was not DE in the AM or MTG (Figure 3b), demonstrating brain region specificity of its modulation. Importantly, a similar increase was also observed in the SN model prior to correction for cell‐type markers (1.7‐fold increase, *p* < .001; full uncorrected model lncRNA results in Table S5). We further found a 1.8‐fold increase in SNCA overexpressing compared to control MPNCs (Figure 3e), suggesting evolutionary conservation of its PD‐related role in mammalian neurons. At the cellular level, LINC‐PINT expression is downregulated by inhibition of the cancer‐related protein P53 (Marin‐Bejar et al., 2013) and it interacts with the Polycomb Repressive Complex 2 (PRC2) (Marin‐Bejar et al., 2013, 2017). Changes in LINC‐PINT levels have been identified in cancer (Garitano‐Trojaola et al., 2018), and it has been shown to play a role in myocardial infarction (Zhu et al., 2018) and in embryonic development (Sauvageau et al., 2013). However, to the best of our knowledge, our current study is the first indication for LINC‐PINT function in neurodegeneration.

### 2.4 LINC‐PINT is primarily neuronal, and its levels are modified in development and aging as well as in Alzheimer's and Huntington's diseases

**Figure 4:**
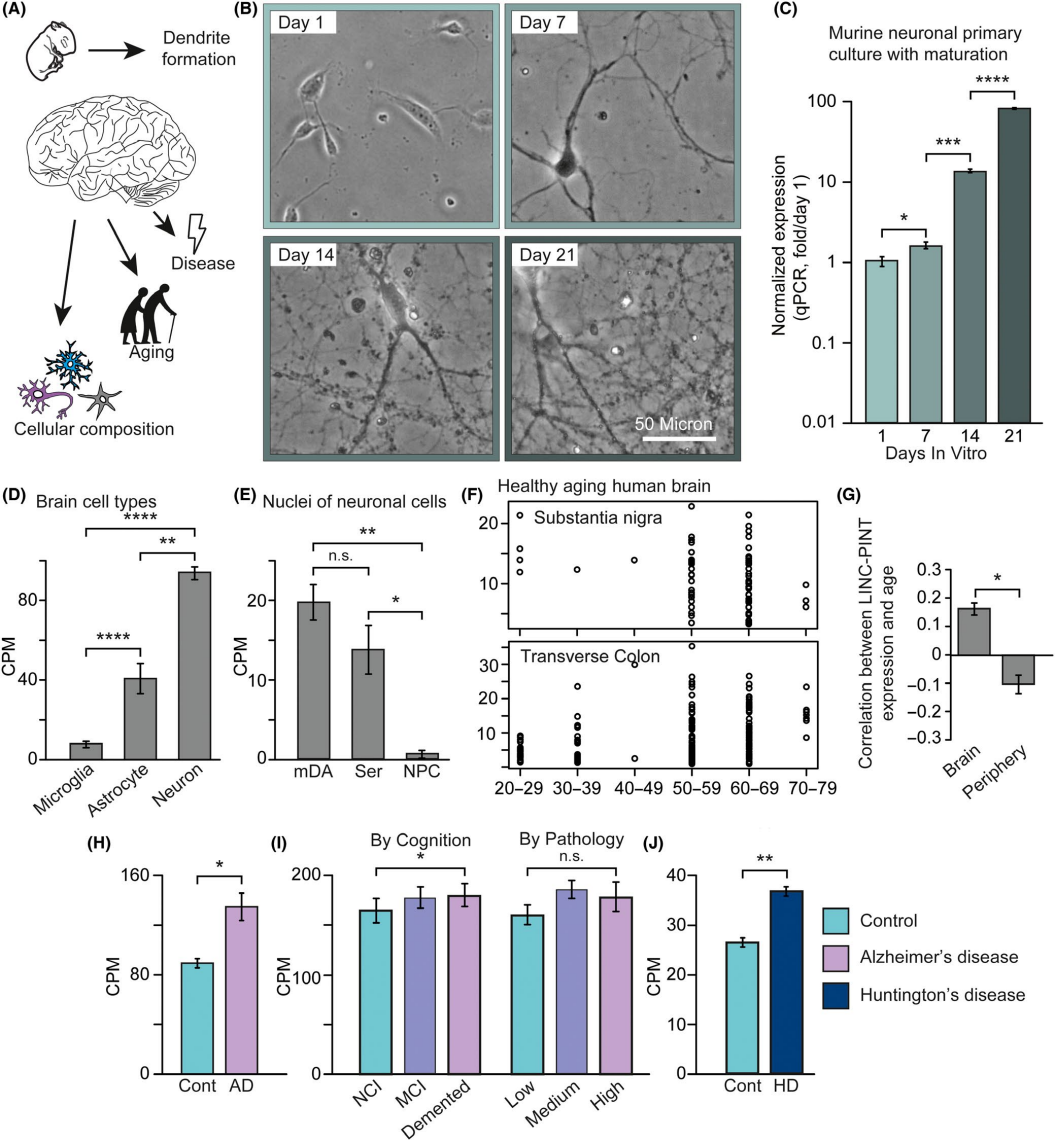
LINC‐PINT is primarily neuronal and correlates with dendrite formation, and its brain expression levels are affected by Alzheimer's disease, Huntington's disease, and aging. (a) LINC‐PINT levels were measured under several conditions associated with brain development and function. (b) Light microscope images demonstrating increased dendritic formation and network complexity with in vitro maturation of MPNCs (Scale bar – 50 μm). (c) Bar plots describing Lncpint expression in maturing MPNCs (*n* = 3 per time point; Welch's *t* test). (d) Bar plots describing Lncpint expression in various FACS‐sorted cell types isolated from the murine brain (adapted from GSE75246; *n* = 4–5 per group; statistical analysis with one‐way ANOVA and Tukey's post hoc analysis). (e) Bar plots describing Lncpint expression in nuclei isolated from various neuronal cell types in the murine brain—midbrain dopaminergic neurons, serotonergic neurons, and neural progenitor cells (adapted from GSE107655; *n* = 3–4 per group; statistical analysis with one‐way ANOVA and Tukey's post hoc analysis). (f) Scatter plots describing the negative correlation between LINC‐PINT levels and age range for Substantia nigra, versus the positive correlation for the Transverse colon (based on the GTEX dataset, see text; *p* < .05 and *p* < .0001, respectively; Pearson's Correlation). (g) Summary of correlation between LINC‐PINT expression and donor age in all brain and peripheral tissues available at GTEX (see Figure S5; Brain: *n* = 13; Periphery: *n* = 35; Welch's *t* test). (h) Bar plots describing LINC‐PINT expression in the lateral temporal lobe of AD, compared to healthy control donors (adapted from GSE104704; *n* = 10 Control, *n* = 12 AD; edgeR analysis, *p* < .05). (i) Bar plots describing LINC‐PINT expression in the temporal gyrus of patients with elevating degrees of pathological and cognitive deterioration (adapted from GSE70424; *n* = 24 per group; edgeR component for cognition—*p* < .05; for pathology—not significant). (j) Bar plots describing the expression of LINC‐PINT in the motor cortex of HD patients compared to control donors (adapted from GSE64810; *n* = 49 Control, *n* = 20 HD; *p* < .01, edgeR analysis)

To test for the role of LINC‐PINT in a plethora of conditions (Figure 4a), including developing and adult neurons, we first measured the levels of its murine homolog, Lncpint, at several stages during the in vitro maturation of MPNCs derived from the cortex of mouse embryos. MPNCs are known to develop more complex dendritic networks over the course of time following their initiation (Lesuisse & Martin, 2002), as could also be seen in our imaging data (Figure 4b). Intriguingly, Lncpint levels were dramatically elevated, by nearly 80‐fold during 21 days in culture (Figure 4c). Interrogating web‐available data of FACS‐sorted neurons, microglia, and astrocytes from murine brains (GSE75246) further revealed that Lncpint levels are highest in neurons and lowest in microglia (Figure 4d), suggesting the elevation of Lncpint levels in maturing primary cultures, as well as in the PD brain, originates from neuronal expression, and not from expression in other cell types. In addition, Lncpint showed elevation in the nuclei of both adult dopaminergic and serotonergic neurons compared to neural progenitor cells (Figure 4e, GSE107655), demonstrating further relevance to PD and to neuronal differentiation.

Since neurodegeneration is an age‐related phenomenon, we next sought association of LINC‐PINT levels with aging in the genotype‐tissue expression (GTEX) human database (Mele et al., 2015). This project incorporates expression data from multiple tissues of over 100 individuals, and analysis of its data identified discordance in LINC‐PINT levels between different brain and body regions. Specifically, examination of the SN displayed age‐correlated depletion of LINC‐PINT, whereas peripheral tissues, such as the transverse colon, displayed an age‐correlated increase (Figure 4f). To systematically test this difference, we examined all tissues which had over 50 samples—overall 48 regions, 13 of which were brain regions (Figure S5). Overall, brain regions displayed decreased levels of LINC‐PINT with advancing age, whereas nonbrain tissues demonstrated an opposite effect (Figure 4g)*.* Thus, the PD‐related SN increases in LINC‐PINT are inverse to its age‐related decreases, which may precede PD by decades in the neurologically normal brain.

Recent research has suggested the involvement of several common genetic and molecular pathways in multiple neurodegenerative conditions (Gan, Cookson, Petrucelli, & Spada, 2018). To test the relevance of this phenomenon for lncRNAs at large and for LINC‐PINT specifically, we measured the expression of LINC‐PINT in AD and HD by investigating brain expression data from WADs of effected individuals and neurologically normal controls. We identified a 45% elevation of LINC‐PINT in the lateral temporal lobe of AD patients compared to healthy donors (Figure 4h, GSE104704) (Nativio et al., 2018). LINC‐PINT elevation in AD was also reproduced in our own RNA‐Seq cohort (Barbash, Garfinkel, et al., 2017), where patients were divided into sub‐groups based on their cognitive impairment and their brain pathology. Intriguingly, LINC‐PINT elevation was correlated with increased cognitive impairment but not with exacerbated brain pathology (Figure 4i, GSE70424). In addition, we also identified an elevation of LINC‐PINT in the motor cortex of HD patients (Figure 4j, GSE64810) (Labadorf et al., 2015). Combined, these results suggest a complex expression pattern for LINC‐PINT in the brain, which is neuron‐centric, elevated with dendrite development and in advanced neurodegeneration on the one hand, and downregulated with age on the other hand.

### 2.5 Tissue culture and murine models display PRC2 links and an oxidative stress‐inducible neuroprotective role for LINC‐PINT

**Figure 5:**
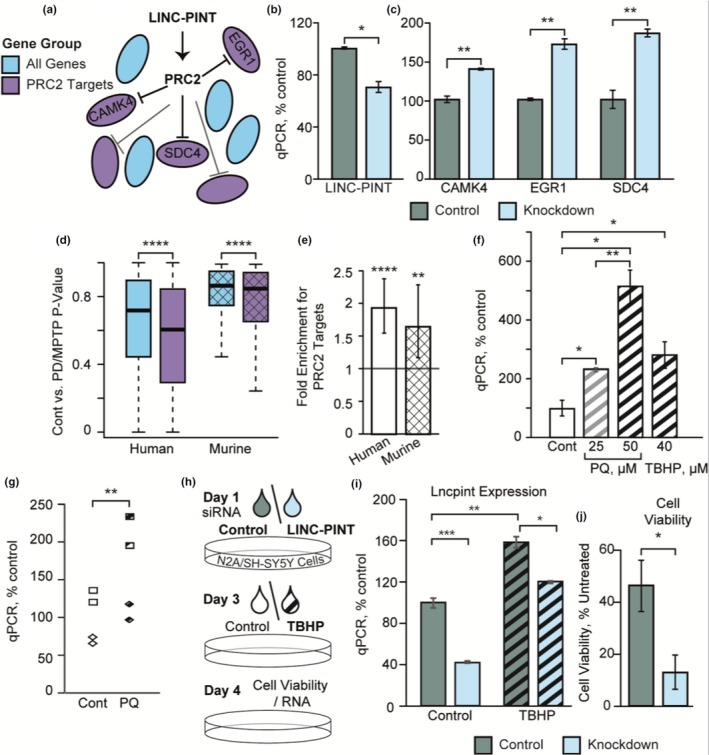
LINC‐PINT associates with PRC2 target expression, and induction of its elevation by oxidative stress has a neuroprotective effect. (a) Scheme displaying the interaction between LINC‐PINT, PRC2, and PRC2 targets. (b + c) Bar plots displaying the effect of LINC‐PINT knockdown using GapmeRs in SH‐SY5Y cells on the expression of LINC‐PINT and three selected PRC2 targets (*n* = 2 for control; *n* = 4 for knockdown; Welch's *t* test). (d) Box plots of FDR‐corrected *p*‐values for PD (in the human SN) and for MPTP treatment (in murine brain‐derived dopaminergic neurons; GSE54795), separated to targets of PRC2 and other genes (*p* < .0001, Mann–Whitney U test). (e) Bar plots displaying the fold enrichment of downregulated genes among PRC2 targets in both models described in (d) (Fisher's exact test). (f) qPCR‐measured LINC‐PINT mRNA levels in control, 25 μM and 50 μM PQ‐treated, and 40 μM TBHP‐treated SH‐SY5Y human neuroblastoma cells (*n* = 2–4 per group, Welch's *t* test). (g) qPCR‐measured Lncpint mRNA levels in control and 2.5 μM PQ‐treated MPNCs of cortical (rhombus) and striatal (square) origin (*p* < .01, two‐way ANOVA for treatment and brain region). (h) Scheme outlining the experimental procedure of the qPCR and MTT assays described in J‐K and Figure S7. (i) qPCR‐measured murine LINC‐PINT homolog Lncpint mRNA levels in cells treated with control or Lncpint‐targeting siRNA pools, with or without 20 μM of TBHP (*p* < .01 for TBHP‐induced elevation with control siRNA, *p* < .001 for Lncpint depletion without TBHP treatment, and *p* < .05 for Lncpint KD with TBHP treatment; *n* = 3 for conditions without TBHP treatment, *n* = 2 for conditions with TBHP treatment; Welch's *t* test). (j) Cell viability compared to non‐TBHP‐treated N2A cells is diminished by Lncpint depletion in 40 μM TBHP‐treated cells (*n* = 6; *p* < .05, Welch's *t* test)

Reports of LINC‐PINT/PRC2 interaction (Marin‐Bejar et al., 2013) argue a role for LINC‐PINT in transcriptional repression of multiple genes (Scheme in Figure 5a)(Kennerdell, Liu, & Bonini, 2018; von Schimmelmann et al., 2016). To test for the relevance of this interaction in our cell model, we used a WAD of neuroblastoma cells treated with the EZH2‐inhibitor GSK126 to extract a list of target genes of PRC2 (GSE85431, see list in Table S6) (Chen et al., 2018). Among those, the three DE transcripts which exhibited the most significant decrease in the SN of PD patients in our dataset were syndecan‐4 (SDC4), calcium/calmodulin‐dependent protein kinase IV (CAMK4), and early growth response 1 (EGR1). To test whether this decrease could be mediated by LINC‐PINT, we modeled its neuronal knockdown by administering LINC‐PINT‐targeted GapmeRs (Qiagen) to the human neuroblastoma cell line SH‐SY5Y. This treatment caused a 30% decrease in LINC‐PINT levels compared to control GapmeRs (Figure 5b) and led to an increase in the levels of all three examined PRC2 targets (Figure 5c). Next, we performed a systemic analysis of all identified PRC2 targets in both our SN dataset and a WAD of murine midbrain dopaminergic neurons extracted from mice treated with 1‐methyl‐4‐phenyl‐1,2,3,6‐tetrahydropyridine (MPTP) or with saline (GSE54795) (Brichta et al., 2015). The FDR‐corrected *p*‐value of MPTP exposure or PD was lower for PRC2 targets compared to other genes (Figure 5d); also, PRC2 targets were enriched among downregulated genes (1.6‐fold enrichment for MPTP, 1.9‐fold enrichment for PD; Figure 5e). Given the literature on LINC‐PINT function, these results suggest that the LINC‐PINT/PRC2 interaction, identified in cancer, is also relevant in the context of brain and neurodegeneration.

The elevation of LINC‐PINT across a plethora of neurodegenerative conditions indicated a common pathway, such as oxidative stress, which is highly relevant in PD (Hauser & Hastings, 2013). To challenge this prediction, we exposed SH‐SY5Y cells to elevating doses of PQ and to stable peroxide tert‐butyl hydroperoxide (TBHP), which induced dose‐dependent, 2.3‐fold to fivefold elevation of LINC‐PINT levels (Figure 5f). Having shown that 21‐day‐old MPNCs express high levels of Lncpint, we tested the effect of oxidative stress on these cells as well. Using 21‐day‐old MPNCs extracted from both fetal cortex and striatum, we observed a 1.5‐ to 1.6‐fold increase in Lncpint levels in both regions after PQ exposure (Figure 5g). Likewise, interrogating a WAD of RNA‐Seq of the striatum of mice exposed to PQ (GSE36232) (Gollamudi et al., 2012) revealed a 5.5‐fold increase in Lncpint levels in the exposed mice (Figure S6), demonstrating parallel effects of oxidative stress at the tissue level. This indicated functional involvement of both LINC‐PINT and its murine homolog in neuronal oxidative stress responses.

LINC‐PINT’s accumulation under exposure to oxidative stress could potentially reflect a reaction which attenuates disease progression and assists cellular survival. Alternatively, LINC‐PINT increases might merely reflect disease pathophysiology or be a side effect of the cellular response to oxidative stress, mediating no impact on cell survival. To understand the functional role of LINC‐PINT and thereby distinguish between these options, we used the murine neuroblastoma cell line N2A to model Lncpint knockdown through the use of siRNA pools (siTools) which specifically target it with minimal off‐target effects. We also induced oxidative stress through TBHP exposure (Experimental scheme in Figure 5h). TBHP induced a 1.6‐fold Lncpint elevation in N2A cells; exposure to siRNA targeting Lncpint reduced its levels in N2A cells by 58% compared to treatment with control siRNA in cells not exposed to TBHP, and to a lesser degree in TBHP‐treated cells (Figure 5i). Next, we used the MTT assay to estimate the impact of Lncpint knockdown on cell viability. Exposure of cells to TBHP resulted in exacerbated cell death under Lncpint knockdown, with only 15% compared to 45% viable cells remaining following TBHP exposure compared to untreated control cells, marking a 66% decrease in viable cells (Figure 5j); this effect was replicated, albeit to a lesser extent (20% decrease in cell viability) using a second, less efficient set of Lncpint‐targeting siPools which generated a 35% depletion of Lncpint (Figure S7). Following the same protocol with SH‐SY5Y cells showed a similar, although weaker, effect on cell viability, with a mere 15% decrease in viable cells (Figure S7). Together, these results demonstrated that Lncpint and LINC‐PINT downregulation consistently jeopardized cellular survival following application of oxidative stress, indicating a neuroprotective role for LINC‐PINT and suggesting that its elevation in PD and its models may be neuroprotective.

## 3 DISCUSSION

Our study has established the first RNA‐sequencing resource of brain tissues from PD patients and control donors. We have sequenced 65 samples from three brain regions, SN, AM, and MTG, and identified differential expression of numerous coding and noncoding genes. Of those, a large set of lncRNAs was specifically expressed in the SN but not in other brain regions. The fraction of SN‐specific lncRNAs was six‐fold higher than that of protein‐coding genes. While many of the SN‐specific lncRNAs were expressed at low levels, some of them were DE, together marking the uniqueness of lncRNA expression patterns in the SN and indicating an important role for lncRNAs in the PD‐effected SN.

To improve our prospects of identifying functionally important lncRNAs, we employed a dual‐parameter statistical analysis, the combined calculated score (CCS), which in other systems identified functionally important lncRNAs in a reproducible manner. This analysis detected SN‐specific dysregulation of several lncRNAs in PD, including the neurogenesis‐implicated RMST, the paraspeckle‐essential NEAT1, and the P53‐regulated LINC‐PINT (Marin‐Bejar et al., 2013; Wan et al., 2017). Next, we considered that even following the correction by cell‐type‐specific confounders, the altered SN lncRNA profile could reflect cellular composition changes rather than a transcriptional and functional modification in particular cell types. This was crucial since the extracted tissue originated from patients with advanced PD, and changes seen in it might improperly reflect the early stages of PD. To challenge this further prediction, we used WADs of SNCA‐overexpressing MPNCs and identified LINC‐PINT, NEAT1, and RMST as modulated in MPNCs as well.

While not dismissing the importance of additional lncRNAs, our survey marked LINC‐PINT, NEAT1, and RMST as having higher prospects of being functionally relevant to disease pathology. Of those, NEAT1 involvement in PD has recently been demonstrated (Liu & Lu, 2018), further supporting the notion that our analysis pipeline identifies functionally relevant lncRNAs. To provide yet another filter for false‐positive results, we performed qPCR tests of RMST, NEAT1, and LINC‐PINT in a larger cohort of patients’ brain samples. This further test marked the elevation of RMST as nonrobust and identified LINC‐PINT as a leading transcript that is prominently changed in the PD SN; measurements of LINC‐PINT’s murine homolog, Lncpint, through the maturation of MPNCs in vitro revealed its prominent elevation, alongside dendritic growth and increased network complexity. In healthy donors, large‐scale transcriptomic data from the GTEX database (Mele et al., 2015) revealed age‐related decreases in LINC‐PINT expression in several key brain regions, including the SN. Combined, these findings provided preliminary evidence for the involvement of this lncRNA, previously identified as involved in cancer (Garitano‐Trojaola et al., 2018), myocardial infarction (Zhu et al., 2018), and embryonic development (Sauvageau et al., 2013), and in neurodevelopment and neurodegeneration as well.

Interrogation of both in‐house and web‐available cell culture and murine model datasets of PQ‐ and TBHP‐induced oxidative stress confirmed upregulation of LINC‐PINT by qPCR and RNA‐Seq. Furthermore, web‐available data of transcriptomic patterns in neurodegeneration revealed increases in LINC‐PINT expression in HD and AD, where this increase correlated with cognitive decline but not with the brain's pathological hallmarks, suggesting that it is not merely a marker of tissue damage. To further pursue the role of LINC‐PINT upregulation in neurodegeneration, we exposed cultured cells of murine origin to TBHP‐induced oxidative stress while using siRNA pools to knockdown the expression of Lncpint. Lncpint depletion exacerbated oxidative stress‐induced cell death under TBHP exposure. This effect was reproduced, although to a smaller scale, using a second less efficient set of siPools in N2A cells, and also in LINC‐PINT depleted human‐originated SH‐SY5Y cells.

That LINC‐PINT depletion exacerbated TBHP‐induced cell death indicated that LINC‐PINT has a neuroprotective role against oxidative stress, also raising the possibility that elevation of LINC‐PINT in the PD brain and upon exposure to oxidative stress may be protective. LINC‐PINT elevation in the brain of PD, AD, and HD patients further marks it as part of a putative neuroprotective mechanism common to multiple aging‐related neurodegenerative processes. This may relate to LINC‐PINT’s interaction with PRC2 (Marin‐Bejar et al., 2013, 2017), which represses genes involved in cell death in general, and in neuronal cell death specifically (von Schimmelmann et al., 2016). Our experiments and analysis also revealed increased levels of PRC2 targets in LINC‐PINT depleted cells and decreased levels in the PD SN, as well as in dopaminergic neurons from MPTP‐exposed mice, further supporting the relevance of the LINC‐PINT/PRC2 interaction in the PD brain. Such a common neuroprotective pathway may be useful to consider, both in the future research of neurodegeneration and in the search for strategies to delay the onset and progression of PD and other neurodegenerative diseases.

## 4 MATERIALS AND METHODS

### 4.1 Brain samples

Frozen human brain tissues were obtained from the Netherland's Brain Bank (NBB) and approved for use by the NBB and by the ethics committee of the Hebrew University (for full list of samples, see Table S1). Diagnosis of donors with PD was done ante‐mortem, according to clinical criteria. For RNA extraction, tissue pieces were cut on dry ice and snap‐frozen in liquid nitrogen to maintain RNA integrity. Lysis and homogenization (using a pellet pestle) were performed with 700 μl QIAzol Lysis Reagent, after which homogenates were snap‐frozen again, before RNA extraction with the miRNeasy Kit (both from Qiagen).

### 4.2 Cell culture

SH‐SY5Y and N2A cells were both grown in standard conditions, according to the ATCC guidelines, using reagents from Biological Industries (Israel). Growth medium for SH‐SY5Y cells was a 1:1 mixture of EMEM and HAM's F12 nutrient mixture, supplemented with 10% FCS, 1% l‐glutamine, and 1% PSA. Growth medium for N2A cells was EMEM supplemented with 10% FCS, 1% l‐glutamine, and 1% PSA. Cells were grown at 37°C and in 5% CO_2._

### 4.3 RNA extraction from cell culture samples

Medium was aspirated, and wells were washed once with PBS, followed by cell lysis and homogenization in 700 μl QIAzol. RNA was extracted using the miRNeasy kit according to kit instructions and treated with Ambion DNAse (Thermo Fisher Scientific).

### 4.4 qPCR

cDNA was prepared using the Quanta qScript mRNA cDNA Synthesis Kit (Quantabio) according to manufacturer's instructions and diluted 1:10 in double‐distilled water prior to qPCR plate preparation. qPCR was performed in either 384‐well or 96‐well plates, using PerfeCTa SYBR Green FastMix Low or no ROX (Quantabio), respectively, at a final well volume of 5 or 15 μl, respectively. TUBB3 was used as a housekeeping gene for brain samples and N2A cells. TUBB3 and RPL19 were used as a housekeeping gene for SH‐SY5Y cells. Expression was calculated as ΔΔCt values (primer sequences are detailed in Table S7).

## CONFLICT OF INTEREST

The authors report no conflict of interests.

## AUTHOR CONTRIBUTIONS

AS planned and performed most experiments; MH constructed RNA‐Seq libraries; NY and SL performed experiments in primary neuronal culture; HS and SK provided scientific guidance; AS and HS wrote the text, ERB and DG edited it, and all co‐authors read and approved the final version.

## Data Availability

Data were submitted to the gene expression omnibus (GEO), where it is available as GSE114517.
