# Supplementary material for: A lncRNA survey finds increases in neuroprotective LINC‐PINT in Parkinson’s disease substantia nigra
Source: Aging Cell. 2020 Feb 20;19(3):e13115. doi: 10.1111/acel.13115 (PMC7059180; doi:10.1111/acel.13115)
Supplement: Supplementary file 1 [file ACEL-19-e13115-s001.docx]

**Supplementary Materials and Methods**

**Study Design** – The study was aimed at discovering disease-related lncRNAs, by using ribosomal RNA-depleted libraries for RNA-Seq to screen lncRNAs in the SN of PD patients compared to non-demented controls, with additional brain regions serving to seek region-specificity of these findings. Subsequent cell culture experiments were performed to understand the mechanisms by which LINC-PINT might be involved in PD neuropathology. In these tests, growth time, calibration of reagent concentrations and length of exposure served to assess the maximal impact of such interventions. Each experiment was repeated at least thrice. Further analysis of published web-available data was then performed to challenge and validate our findings. In all analyses, outliers beyond two standard deviations from the mean were considered invalid and were removed.

**Knockdown experiments** – 24 hours prior to transfection, 96- or 12-well plates were plated with 50 or 500 thousand N2A cells/well. Transfection of N2A cells was performed with siRNA pools consisting of 30 different siRNAs (siTOOLs Biotech) at a total final concentration of 10 nM siRNA using Lipofectamine*®* RNAiMax Transfection Reagent (Invitrogen) according to manufacturer’s protocol, for maximum Lncpint-directed knockdown and minimal off-target effects (Hannus et al., 2014). Transfection of SH-SY5Y cells was performed using GapmeRs (Qiagen) at a final concentration of 75 nM using HiPerfect transfection reagent (Qiagen). 150 pico-mol were added to 175 μL of EMEM, supplemented by 25 μL transfection reagent and incubated for 5 minutes before added drop-wise to a 6-well cell culture plate. TBHP (Sigma-Aldrich), dissolved in double-distilled water (DDW), or DDW-only control, were added 48 hours post-transfection.

**RNA-Seq library preparation** – Libraries were prepared using the RNA-Tag Seq method (Shishkin et al., 2015). Ribosomal RNA depletion was performed using the Ribo Zero Gold rRNA removal kit (Illumina), with residual ribosomal RNA fractions of 7-9%. Libraries were then run using the NextSeq® 500 High Output v2 Kit (75 cycles) on the NextSeq 550 System (both from Illumina). Data was uploaded to the GEO (GSE114517).

**RNA-Seq analysis** – FASTQ files were uploaded to the Galaxy server (Afgan et al., 2016). Reads were aligned to the hg38 version of the human genome using HISAT2 (Kim et al., 2015). PCR-induced duplicated reads were removed using Rmdup from the SAMtools package (Li et al., 2009). Counts were obtained using Htseq (Anders et al., 2015) and the Gencode23 genome annotation (Harrow et al., 2012). Differential expression was determined in R using the edgeR package (Robinson et al., 2010), after excluding 10 out of 75 libraries with fewer than 0.5 million reads (6 SN; no AM; 4 MTG; this elimination was further based on post-analysis lack of credibility due to lack of change in known PD-related genes). The differential expression model was calculated separately for each tissue, and included several parameters – age, gender, RIN, and disease status for all samples. In addition, SN samples were adjusted for additional confounders, based on the expression of cell-type markers – the astrocyte marker ALDH1L1, the microglia marker TMEM119 and the dopaminergic neuron marker TH – thus making sure identified changes were not caused by changes in SN tissue histology, which is a prominent mark of PD (Fearnley and Lees, 1991). To further verify the validity of these confounders, we extracted from the model genes whose differential expression correlated with these three biomarkers, and examined the expression patterns of known cell-type marker transcripts in correlation with these confounders. This was done by identifying genes which are specifically expressed in these different cell types, according to web-available data from a recently published work in mice (GSE75246; Gene list in Table s8; Scheme in Fig. s2A) (Srinivasan et al., 2016). Compatible with our prediction, we found that these cell-type specific genes (which showed count per million (CPM)>1 expression for only a single cell type) present larger changes than those of other brain-expressed genes by their corresponding cell-type confounder (Fig. s2B). Correction for multiple comparisons by false discovery rate (FDR) calculation, as well as comparison of protein-coding genes and lncRNAs, was performed after removal of genes with logCPM<1. LncRNA differential expression score was calculated using the following formula: $Score =log(2^{logCPM}/(p-value))$.

Primary steps to ensure the validity of this method appear in the Results section. To further challenge the capacity of our approach for exploring RNA-Seq sources for lncRNA differences, we analyzed three very diverse web-available pairs of datasets, including Monocyte stimulation with LPS (GSE107821 and GSE111927) (Crisan et al., 2017), ARID1A gene knockdown (GSE119779 and GSE114576) (Livshits et al., 2018; Wang et al., 2018) and Huntington’s disease neural stem cells (GSE74201 and GSE95343) (Consortium, 2017; Ring et al., 2015). Treating one set of each pair as the training dataset and the second as the validation dataset enabled differential expression analysis, and we used the analysis of the training set to identify two groups of lncRNAs – those with highest scores and those with smallest p-values. In all three data set pairs, comparison of these lncRNA groups in the validation dataset revealed lower p-values for the group of highest-scoring lncRNAs (Analysis scheme in Fig. s3A and results Fig. s3B).

**Murine primary neuron culture** – On day E14.5, mice were sacrificed and mouse embryo heads were isolated on ice. The outer cuticle was removed, following by removal of the brain with curved forceps. Cortex or striatum were isolated and transferred to a 1.5 mL vial with Hank’s Balanced Salt Solution (HBSS, Biological Industries). After isolation of all embryo heads, supernatant was aspirated and brains were chopped after addition of new ice-cold HBSS. Tissue was incubated until it sunk, supernatant was aspirated and new HBSS was added. This was repeated twice overall. After second aspiration, Papain Solution was added (for 3 embryo brains: 3 mL PBS with 0.6 mg L-Cystein Hydrochloride; 25 mg D-Glucose; 1 mg BSA; 1500U DNAse I; All by Sigma-Aldrich; 60 U Papain - Worthington), following by a 15-minute incubation at 37°C, with invertion of the tubes every 3-5 minutes. A double volume of Plating Medium was added to stop digestion (Alpha-MEM – Biological Industries - supplemented with 5% FBS, 0.6% w/v D-glucose, GlutaMax© 1:100, Antibiotic-Antimycotic 1:100 – Life Technologies), following by discarding of supernatant. Pellet was mixed well with 1 mL of DNAseI Solution per 3 embryos (HBSS supplemented with 31.5 mM Magnesium Chloride and 500 U/mL DNAse I – Sigma-Aldrich), pipetted up and down multiple times and passed through a 70 μM Falcon cell strainer (BD Biosciences). Mixture was centrifuged at 1200 RPM for 5 minutes, supernatant removed and pellet resuspended in 10 mL Plating Medium. This was repeated twice. Cells were then counted and plated on Poly-L-Lysine (Sigma-Aldrich) coated dishes at the predetermined concentration. After 3-6 hours, medium was changed to pre-warmed culturing medium (Neurobasal© medium supplemented with: B27© 1:50; GlutaMax© 1:100 and Antibiotic-Antimycotic 1:100 – all by Life Technologies). Cells were grown and medium changed every 3 days – half the medium was changed, and supplemented with Cytosine Arabinoside (Sigma-Aldrich) at the final concentration of 1 μM to inhibit proliferation of glial cells**.**

**Web-available GEO datasets** – Human data was acquired from the Gene Expression Omnibus (GEO). Analysis was performed using Galaxy. For murine samples we used the M15 version of the GENCODE annotation. A full list of datasets used, including their description, origin and group definitions, are detailed in Table s9.

**GTEX Dataset** – restricted donor age data from the phs000424.v7 version of the Genotype-Tissue-Expression (GTEX) project (Mele et al., 2015) was acquired via the dbGap system after proper authorization. Pearson’s correlation was used to correlate between donor age and LINC-PINT expression (measured in CPM). In order to comply with the data restriction policy, data displayed in Figure 4F was de-identified and displayed based on the publically-available age category; however, p-values and correlation estimates are based on the exact, restricted age data.

# MTT cell viability assay – The assay was carried out in 96-well plates, 24 hours after TBHP administration. Cell medium was supplemented with 20 μL of 5 mg/mL MTT (Thiazolyl Blue Tetrazolium Bromide, Sigma-Aldrich) in PBS, followed by 4 hours of incubation at 37°C and 5% CO_2_. Salt crystals were then dissolved by adding 100 μL 10% SDS containing 0.01N HCl and over-night incubation at 37°C. Absorptions were read at 570 nm for MTT and 690 nm for reference using a Spark 10M Tecan microplate reader.

**Statistics** – Statistical analysis was performed using R. For two-group comparisons we first used a Kolmogorov-Smirnoff test to determine whether the data showed a normal distribution. If it did, we performed Welch’s t-test and if not, we used the Mann-Whitney’s U test. For comparison of absolute log fold change values, we used bootstrapping to assess confidence interval, and presented the data in bar plots. Normally distributed data was presented in bar plots, with error bars marking the standard error. Other data was displayed as either a cumulative frequency plot or a boxplot, in which the line represents the median, box limits represent the 25^th^ and 75^th^ percentiles and the whiskers extend to the farthest data point which is at a maximum of the 25^th^ to 75^th^ percentile distance from the edge of the boxplot. Data on primary culture from two origins was subjected to two-way ANOVA analysis.

**Supplementary Figures**

**Figure S1:**

**
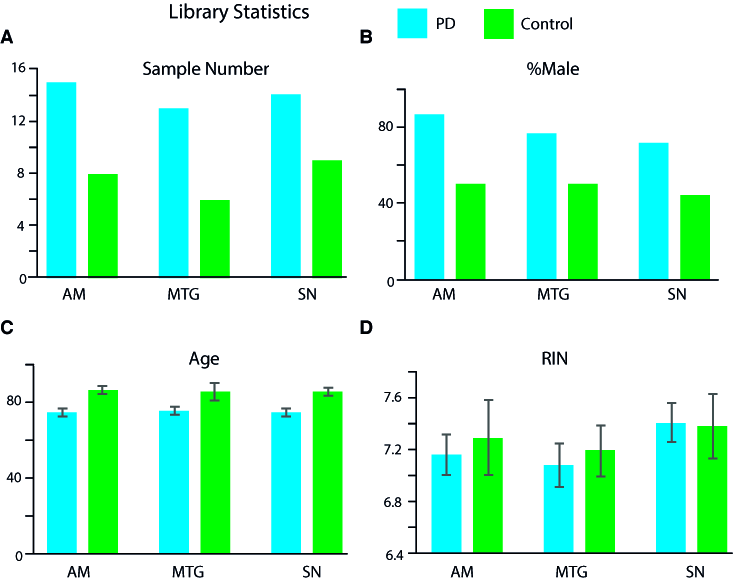
**

**Demographic and technical parameters of RNA-Seq samples included in analysis.** Data is presented in bar plots, compared between groups based on different brain regions and between PD patients (teal) and non-PD controls (green). (A) Number of samples per group. (B) Percent of male patients per group. (C) Mean donor age by group (±SD). (D) RNA integrity number values (±SD).

**Figure S2:**


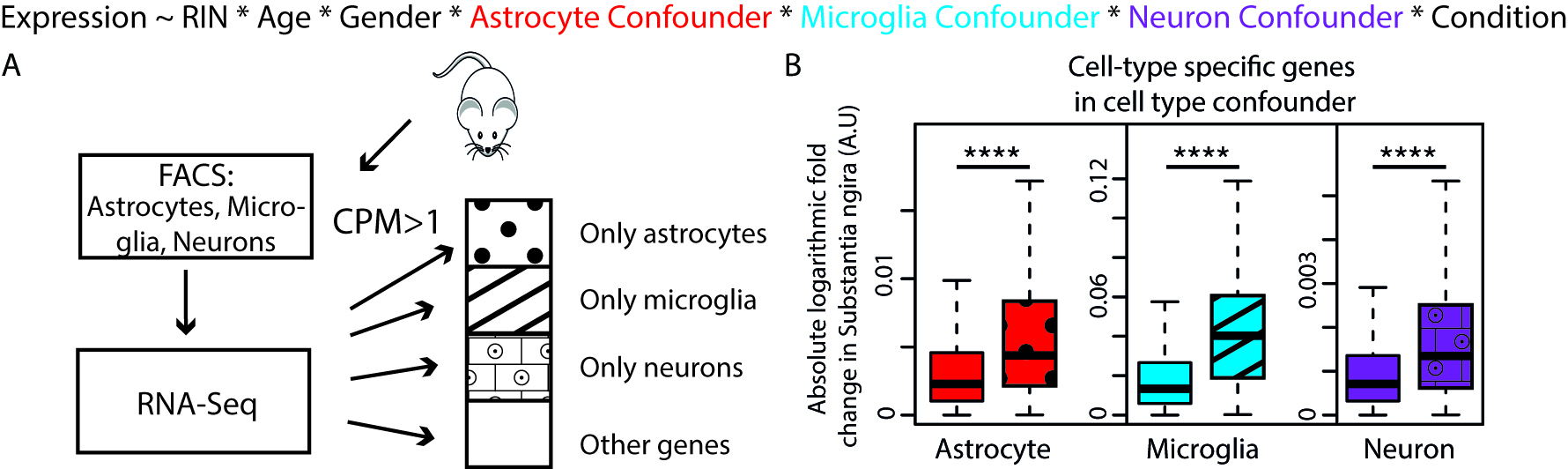


**Pipeline for the determination of cell-type specific factors validity.** (A) Scheme depicting the identification of cell-type specific genes, based on GSE75246. (B) Results of differential expression model components, which are based on the expression of cell type markers – ALDH11L1 for astrocytes, TMEM119 for microglia and TH for neurons. Each panel describes a single cell type marker, with the y axis representing the relative change of each gene in correlation with the specific shown confounder, in arbitrary units. Boxplots describe genes identified (according to panel A) as cell-type specific, vs all other genes (p<0.0001, Mann-Whitney U test).

**Figure S3:**

**
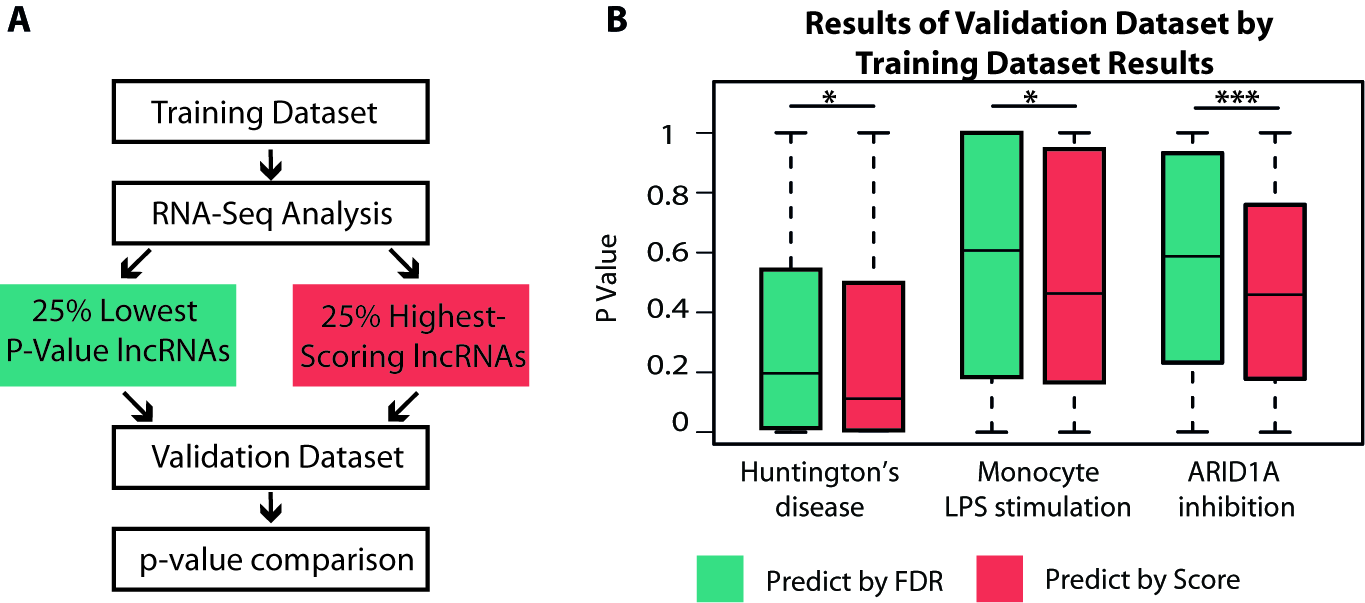
**

**Top-scoring lncRNAs show higher reproducibility than those with lowest p-values in comparison between similar datasets.** (A) Scheme describing the comparisons of top-changing lncRNAs in three pairs of datasets (see table s8 for details). (B) Boxplots of p-values in the validation dataset, based on training dataset FDR/Score (HD – p<0.05; Monocytes – p<0.05; ARID1A – p<0.001; One-tailed Mann-Whitney U test).

**Figure S4:**

**
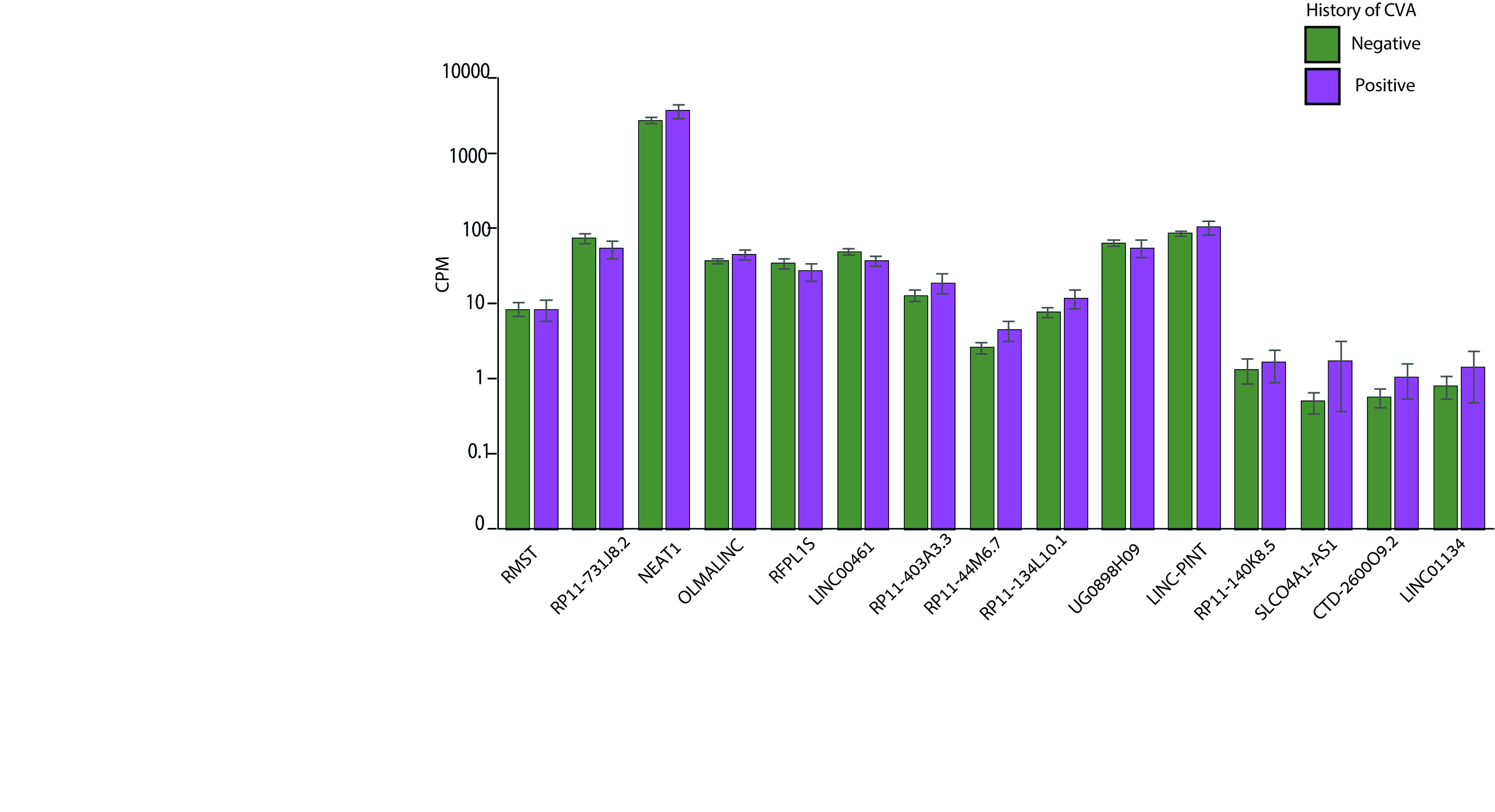
**

**Top-changing lncRNAs are not effected by prior occurrence of cerebrovascular accident (CVA).** Bar plots describing the expression of top-scoring lncRNAs in the SN of patients who have had a prior occurrence of CVA, and those who did not (edgeR analysis; not significant for all comparisons).

**Figure S5:**

**
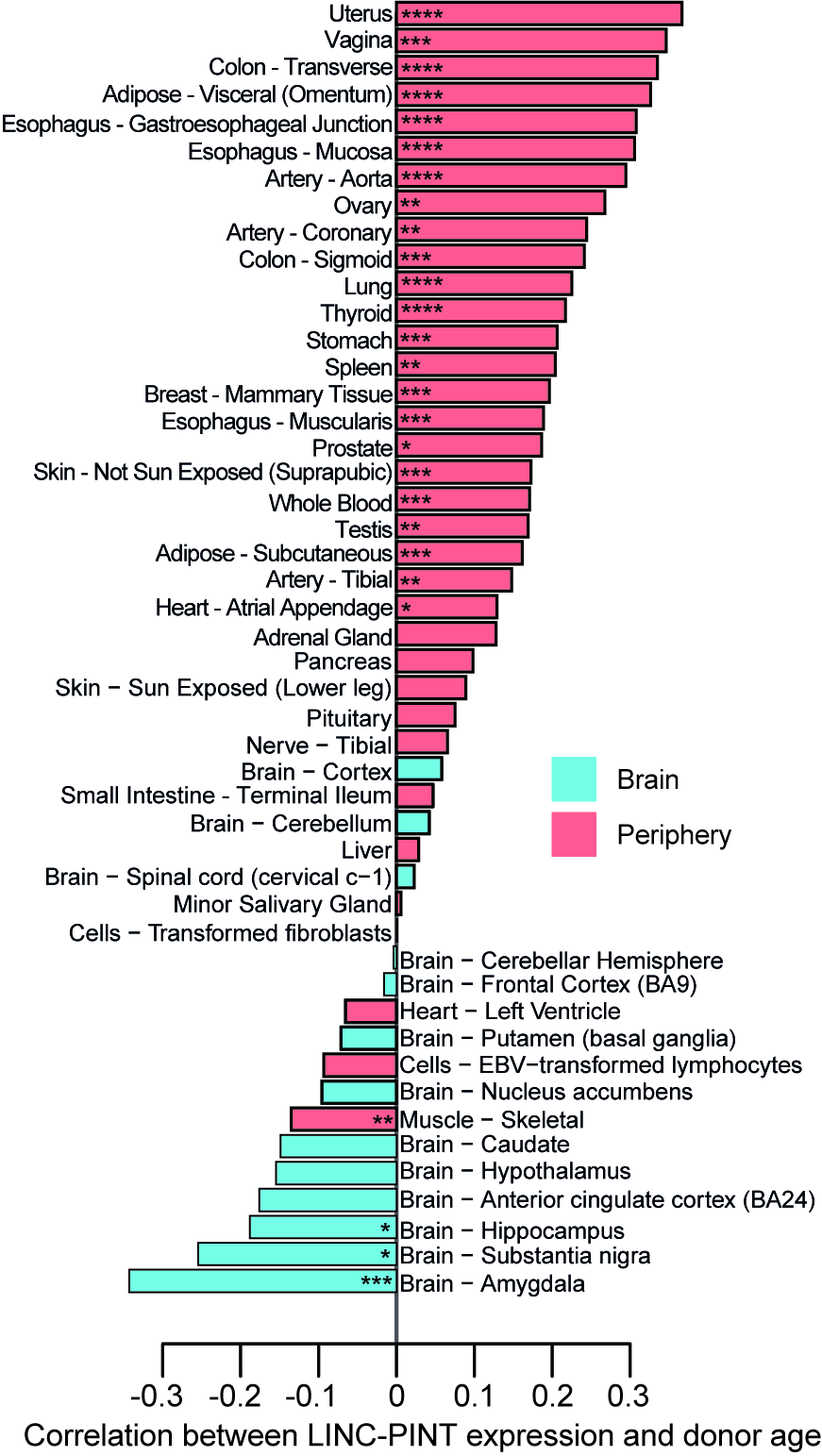
**

**Aging induces changes to LINC-PINT expression across a variety of human tissues.** Bar plots describing the correlation between LINC-PINT expression and donor age, in all GTEX-available tissues; notice netative correlation mostly for brain tissue and positive correlation for extra-brain tissue (p values denoted by Asterix, calculated by Pearson’s test for correlation).

**Figure S6:**

**
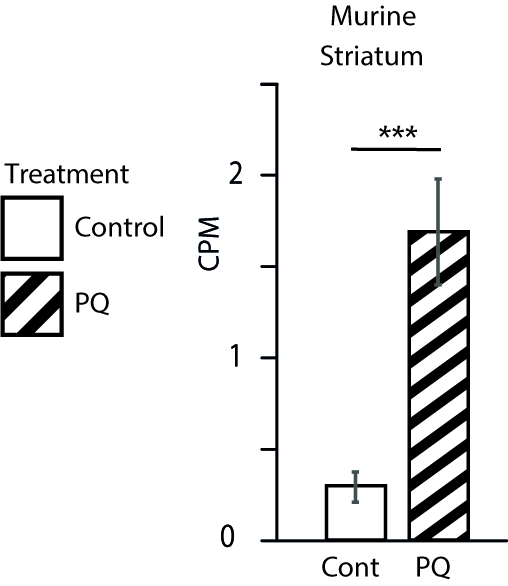
**

**PQ increases Lncpint expression in the murine striatum.** Bar plots representing Lncpint expression in the Striatum of mice treated with PQ (Adapted from GSE36232, n=3 per group, p<0.001, edgeR analysis)

**Figure S7:**

**
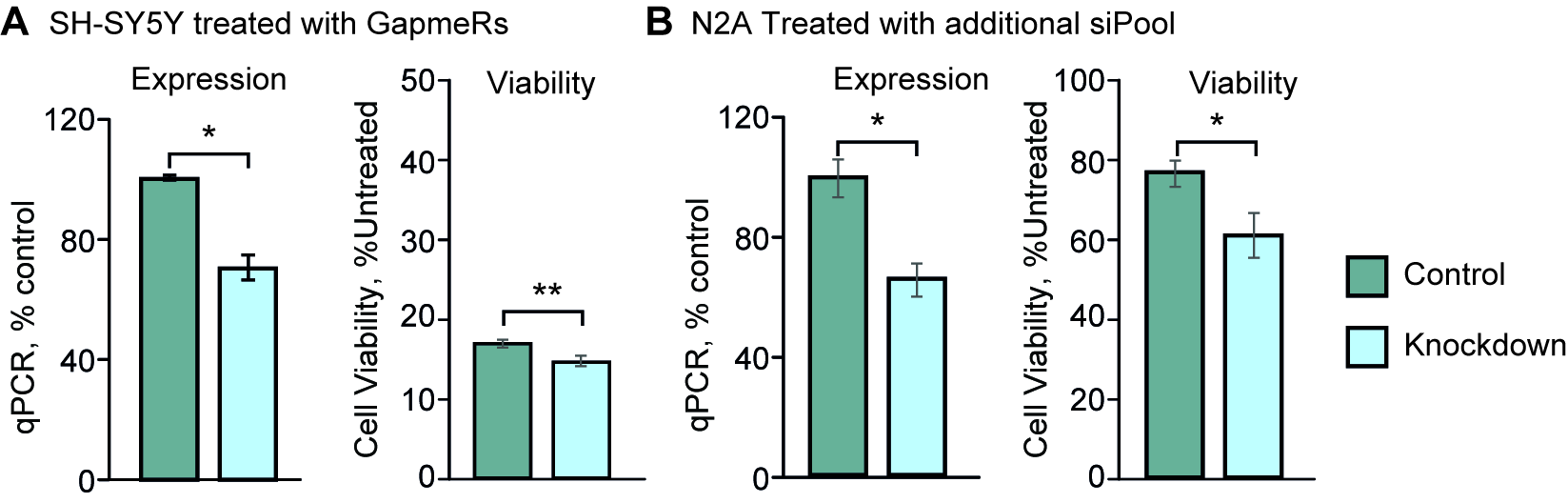
**

**Depletion of LINC-PINT in SH-SY5Y and N2A cells causes an increase in TBHP-induced cell mortality.** (A) GapmeR treatment in SH-SY5Y cells. Bar plots represent Lncpint expression as measured by qPCR (left-hand), and cell viability of cells treated with 40μM TBHP, normalized to non-TBHP treated cells (right-hand). measured by MTT cause a depletion in LINC-PINT levels (Expression: n=2 for control, n=4 for knockdown; p<0.05. Viability: n=24; p<0.01, Welch’s t test). (B) Treatment of N2A cells with a second set of siPools targeting Lncpint, which yielded less efficient knockdown (compared to the main set described in Figure 5). Bar plots as described in panel A (Expression: n=3; p<0.05. Viability: n=22 for control, n=24 for knockdown; p<0.05, Welch’s t test)

**Supplementary Tables**

**S1 Table (as attached file):**

**Basic clinical and demographic data of brain tissue donors from the Netherland’s Brain Bank**. Each row also includes details of the specific use of each sample in the study.

**S2 table (as attached file):**

**SN differentially-expressed genes.** Differentially expressed genes (both protein-coding and noncoding) in the *Substantia nigra* of PD patients, as detailed in the Results and Methods sections.

**S3 Table:**

| Dataset (GEO) | Model | Gene Name | logFC | logCPM | FDR | Score | Location by FDR | Location by Score | Reference for relevance in the model system |
| --- | --- | --- | --- | --- | --- | --- | --- | --- | --- |
| GSE95132 | Colorectal Carcinoma | MALAT1 | 0.988 | 13.14 | 0.018 | 21.28 | 55/85 | 9/85 | (Ji et al., 2014) |
| GSE59288 | Autism | RP11-386G11.10 | 0.326 | 9.776 | 0.035 | 16.66 | 531/603 | 98/603 | (Lin et al., 2014) |
| GSE87534 | Athero-sclerosis | LINC00657 | 0.473 | 8.480 | 0.049 | 14.23 | 187/188 | 98/188 | (Bao et al., 2018) |
| GSE93682 | Neuronal Activity | Meg3 | -1.775 | 10.930 | 0.010 | 21.52 | 77/190 | 12/190 | (Tan et al., 2017) |
| GSE93682 | Neuronal Activity | Rian | -1.584 | 8.534 | 0.015 | 18.24 | 103/190 | 22/190 | (Tan et al., 2017) |

**Application of the dual-parameter analysis paradigm on external RNA-Seq datasets.** Each row describes a single lncRNA gene as it appears in a specific dataset, and includes the identification of the dataset and gene, the results of the edgeR algorithm for this gene, the FDR value and its relative position among all genes, the dual parameter score and its relative position among all genes and a citation suggesting the gene’s involvement in the condition associated with the dataset.

**S4 Table (as attached file):**

**lncRNAs in the SN, AM and MTG and their combined calculated score.** Output of the edgeR algorithm for all SN, AM and MTG expressed (logCPM>1) lncRNAs, combined with gene nomenclature from the Gencode23 annotation, FDR values and their combined calculated scores (CCS).

**S5 Table (as attached file):**

**lncRNAs in the SN – model not corrected for cell type markers.** Output of the edgeR algorithm for all SN expressed (logCPM>1) lncRNAs, combined with gene nomenclature from the Gencode23 annotation and FDR values.

**S6 Table (as attached file):**

**Targets of PRC2/EZH2.** Identified through analysis of web-available RNA-Seq data from two neuroblastoma cell lines treated with EZH2 inhibitor GSK126 (GSE85431).

**S7 Table:**

| Gene Name | Forward Primer | Reverse Primer | Notes |
| --- | --- | --- | --- |
| TUBB3 | GCAACTACGTGGGCGACT | GGCCTGAAGAGATGTCCAAA | Human gene |
| RPL19 | GCTCGATGCCGGAAAAACAC | GCTGTACCCTTCCGCTTACC |  |
| NEAT1 | GGGCCATCAGCTTTGAATAA | GGTGGGTAGGTGAGAGGsTCA |  |
| RMST | TTTTGGACTTTCCCAGGCTA | TTTTGGACTTTCCCAGGCTA |  |
| LINC-PINT | CCTCGCAGATGAGGTAGGAG | TTTTGGACTTTCCCAGGCTA | Human gene |
| Tubb3 | CGCCTTTGGACACCTATTCA | TGCAGGCAGTCACAATTCTC | Murine gene |
| Lncpint | AAACTACGCCACCTCCTCTG | TGAACACTACACCGCTTTGC | Murine gene |
| SDC4 | GGACCTCCTAGAAGGCCGATA | AGGGCCGATCATGGAGTCTT |  |
| CAMK4 | GCCTCGTCCCGGATTACTG | TCCCCTTCTGTTTGCATCTGT |  |
| EGR1 | GGTCAGTGGCCTAGTGAGC | GTGCCGCTGAGTAAATGGGA |  |

**List of all primer pairs used in the study**

**S8 table (as attached file)**

**Cell-type specific genes.** List of genes identified in GSE75246 as being expressed at a level higher than 1 CPM in only one of the three investigated cell types – neurons, microglia and astrocytes, and expressed in the human *substantia nigra*. These genes were used for the calculations in Fig. s2.

**S9 Table:**

| GEO Accession Number | Citation | Origin | Dataset Description | Group Specification |
| --- | --- | --- | --- | --- |
| GSE93682 | Ding et. al, 2017 | murine cortical neurons | Cortical neurons treated with 50mM KCl, causing activation; RNA extracted after 24h | KCl Treated vs. Control |
| GSE59288 | Liu et. al, 2016 | human prefrontal cortex | Post-mortem samples from human prefrontal cortex, taken from donors with autism or control donors | Autism vs. Control |
| GSE95132 | N/A | human colon | Samples taken from KRAS-mutant colorectal carcinoma tumor tissue and adjacent healthy tissue | Tumor vs. Healthy |
| GSE87534 | NA | human umbilical vein endothelial cells | HUVEC cells treated with static vs. slow flow medium, a model for atherosclerosis | Slow vs. Static |
| GSE107821 | Crispan et. al, 2017 | monocytes | Monocytes from healthy donors were exposed to uric acid or control medium for 24h, then to LPS (or control) for 4h | 24h, RPMI treated, Control vs LPS |
| GSE111927 | N/A | monocytes | Umbilical cord monocytes were isolated from healthy newborns or newborns suffering from chorioamnionitis | Healthy unstimulated vs. Healthy LPS |
| GSE119779 | Wang et. al, 2018 | murine pancreas | Pancreas from wild type or pancreas-specific knockdown of Arid1a | WT vs. Arid1a knockdown |
| GSE114576 | Livshits et. al, 2018 | murine pancreatic epithelial cells | Pancreatic epithelial cells sorted from control or conditional Arid1a knockdown mice | Renilla shRNA vs Arid1a shRNA |
| GSE74201 | Ring et. al, 2015 | human iPS-derived neural stem cells | iPSCs from Huntington’s disease (HD) patients or controls were differentiated to neural stem cells (NSCs) | HD vs Control NSCs |
| GSE95343 | Huntington Consortium, 2017 | human iPS-derived neural cells | iPSCs from HD patients or controls were differentiated to neural cells | HD vs Control neural cells |
| GSE70368 | Volakakis et. al, 2015 | Mouse primary midbrain cultures | Overexpression of alpha-synuclein (Snca) with or without transcription factor Nurr1 | Snca vs RFP control |
| GSE36232 | Golamudi et. Al, 2012 | murine ventral midbrain | Several brain regions were taken post-mortem from mice treated with various PD-related toxins | Striatum: PQ vs. Control |
| GSE114517 | In-house | Human brain | Brain tissues from three brain regions | PD+Dementia vs. Non-demented control |
| GSE75246 | Srinivasan et. al, 2016 | Murine brain | Brain tissue was sorted using flow cytometry, based on microglia, astrocytes or neuron markers | CPM data was used for each tissue type (CPM>1 🡪 gene considered expressed in cell type); Also – comparison between expression in three cell types |
| GSE70424 | Barbash et. al, 2017 | Human temporal gyrus | Brain tissue from patients with various degrees of brain pathology and cognitive decline | Dose dependence for cognitive decline and pathology |
| GSE104704 | Nativio et. al, 2018 | Human temporal gyrus | Brain tissue from patients with AD or control donors | Alzheimer’s diseae vs. Aged control donors |
| GSE64810 | Labadorf et. al, 2015 | Human prefrontal cortex | Brain tissue from patients with HD or control donors (mRNA) | Huntington’s disease vs. Control |
| GSE64977 | Hoss et. al, 2015 | Human prefrontal cortex | Brain tissue from patients with HD or control donors (Short RNA) | Huntington’s disease vs. Control |
| GSE107655 | Södersten et. al, 2018 | Murine brain | FACS-sorted nuclei from mice | Comparisons between expression in three nuclei types |
| GSE85431 | Chen et. al, 2018 | Human neuroblastoma cells | Two neuroblastoma cell lines treated with EZH2 inhibitor XX. | Analysis with three variables: Day, Cell Type and Inhibitor Treatment; Results according to the latter. |
| GSE54795 | Brichta et. al, 2015 | Murine dopaminergic neurons | TRAP-isolated dopaminergic neurons from mice treated with MPTP or saline. | MPTP vs. Control |

**List of GEO datasets used in the study**. The list includes references (if available), description and group definitions.

Supplementary References:

Afgan, E., Baker, D., van den Beek, M., Blankenberg, D., Bouvier, D., Cech, M., Chilton, J., Clements, D., Coraor, N., Eberhard, C.*, et al.* (2016). The Galaxy platform for accessible, reproducible and collaborative biomedical analyses: 2016 update. Nucleic Acids Res *44*, W3-W10.

Anders, S., Pyl, P.T., and Huber, W. (2015). HTSeq--a Python framework to work with high-throughput sequencing data. Bioinformatics *31*, 166-169.

Bao, M.H., Li, G.Y., Huang, X.S., Tang, L., Dong, L.P., and Li, J.M. (2018). Long Noncoding RNA LINC00657 Acting as a miR-590-3p Sponge to Facilitate Low Concentration Oxidized Low-Density Lipoprotein-Induced Angiogenesis. Mol Pharmacol *93*, 368-375.

Consortium, H.D.i. (2017). Developmental alterations in Huntington's disease neural cells and pharmacological rescue in cells and mice. Nat Neurosci *20*, 648-660.

Crisan, T.O., Cleophas, M.C.P., Novakovic, B., Erler, K., van de Veerdonk, F.L., Stunnenberg, H.G., Netea, M.G., Dinarello, C.A., and Joosten, L.A.B. (2017). Uric acid priming in human monocytes is driven by the AKT-PRAS40 autophagy pathway. Proc Natl Acad Sci U S A *114*, 5485-5490.

Fearnley, J.M., and Lees, A.J. (1991). Ageing and Parkinson's disease: substantia nigra regional selectivity. Brain *114 ( Pt 5)*, 2283-2301.

Hannus, M., Beitzinger, M., Engelmann, J.C., Weickert, M.T., Spang, R., Hannus, S., and Meister, G. (2014). siPools: highly complex but accurately defined siRNA pools eliminate off-target effects. Nucleic Acids Res *42*, 8049-8061.

Harrow, J., Frankish, A., Gonzalez, J.M., Tapanari, E., Diekhans, M., Kokocinski, F., Aken, B.L., Barrell, D., Zadissa, A., Searle, S.*, et al.* (2012). GENCODE: the reference human genome annotation for The ENCODE Project. Genome Res *22*, 1760-1774.

Ji, Q., Zhang, L., Liu, X., Zhou, L., Wang, W., Han, Z., Sui, H., Tang, Y., Wang, Y., Liu, N.*, et al.* (2014). Long non-coding RNA MALAT1 promotes tumour growth and metastasis in colorectal cancer through binding to SFPQ and releasing oncogene PTBP2 from SFPQ/PTBP2 complex. Br J Cancer *111*, 736-748.

Kim, D., Langmead, B., and Salzberg, S.L. (2015). HISAT: a fast spliced aligner with low memory requirements. Nat Methods *12*, 357-360.

Li, H., Handsaker, B., Wysoker, A., Fennell, T., Ruan, J., Homer, N., Marth, G., Abecasis, G., Durbin, R., and Genome Project Data Processing, S. (2009). The Sequence Alignment/Map format and SAMtools. Bioinformatics *25*, 2078-2079.

Lin, M., Zhao, D., Hrabovsky, A., Pedrosa, E., Zheng, D., and Lachman, H.M. (2014). Heat shock alters the expression of schizophrenia and autism candidate genes in an induced pluripotent stem cell model of the human telencephalon. PLoS One *9*, e94968.

Livshits, G., Alonso-Curbelo, D., Morris, J.P.t., Koche, R., Saborowski, M., Wilkinson, J.E., and Lowe, S.W. (2018). Arid1a restrains Kras-dependent changes in acinar cell identity. Elife *7*.

Mele, M., Ferreira, P.G., Reverter, F., DeLuca, D.S., Monlong, J., Sammeth, M., Young, T.R., Goldmann, J.M., Pervouchine, D.D., Sullivan, T.J.*, et al.* (2015). Human genomics. The human transcriptome across tissues and individuals. Science *348*, 660-665.

Ring, K.L., An, M.C., Zhang, N., O'Brien, R.N., Ramos, E.M., Gao, F., Atwood, R., Bailus, B.J., Melov, S., Mooney, S.D.*, et al.* (2015). Genomic Analysis Reveals Disruption of Striatal Neuronal Development and Therapeutic Targets in Human Huntington's Disease Neural Stem Cells. Stem Cell Reports *5*, 1023-1038.

Robinson, M.D., McCarthy, D.J., and Smyth, G.K. (2010). edgeR: a Bioconductor package for differential expression analysis of digital gene expression data. Bioinformatics *26*, 139-140.

Srinivasan, K., Friedman, B.A., Larson, J.L., Lauffer, B.E., Goldstein, L.D., Appling, L.L., Borneo, J., Poon, C., Ho, T., Cai, F.*, et al.* (2016). Untangling the brain's neuroinflammatory and neurodegenerative transcriptional responses. Nat Commun *7*, 11295.

Tan, M.C., Widagdo, J., Chau, Y.Q., Zhu, T., Wong, J.J., Cheung, A., and Anggono, V. (2017). The Activity-Induced Long Non-Coding RNA Meg3 Modulates AMPA Receptor Surface Expression in Primary Cortical Neurons. Front Cell Neurosci *11*, 124.

Wang, S.C., Nassour, I., Xiao, S., Zhang, S., Luo, X., Lee, J., Li, L., Sun, X., Nguyen, L.H., Chuang, J.C.*, et al.* (2018). SWI/SNF component ARID1A restrains pancreatic neoplasia formation. Gut.

.
